# Cognitive Function and Depressive Symptoms among Chinese Adults Aged 40 Years and Above: The Mediating Roles of IADL Disability and Life Satisfaction

**DOI:** 10.3390/ijerph20054445

**Published:** 2023-03-02

**Authors:** Yixuan Liu, Xinyan Yang, Yanling Xu, Yinghui Wu, Yiwei Zhong, Shujuan Yang

**Affiliations:** Department of Social Medicine and Health Management, School of Public Health, Jilin University, Changchun 130021, China

**Keywords:** cognitive function, depressive symptoms, IADL disability, life satisfaction

## Abstract

The purpose of this study was to investigate the relationship between cognitive function and depressive symptoms among Chinese adults aged 40 years and above, as well as the series of multiple mediating effects of Instrument Activities of Daily Living disability and life satisfaction on this relationship. The data was obtained from the China Health and Retirement Longitudinal Study (CHARLS, 2013–2018), including 6466 adults aged 40 years and above. The mean age of the adults was 57.7 ± 8.5. The SPSS PROCESS macro program was conducted to examine the mediating effects. The results indicated that there was a significant association between cognitive function and depressive symptoms five years later (B = −0.1500, 95%CI: −0.1839, −0.1161), which could also be demonstrated through three mediation pathways: (1) the mediating pathway through IADL disability (B = −0.0247, 95%CI: −0.0332, −0.0171); (2) the mediating pathway through life satisfaction (B = 0.0046, 95%CI: 0.0000, 0.0094); and (3) the chain mediation pathway through IADL disability and life satisfaction (B = −0.0012, 95%CI: −0.0020, −0.0003). Both IADL disability and life satisfaction have been proven to be crucial mediators for the relationship between cognitive function and depressive symptoms five years later. It is necessary to improve individuals’ cognitive function and reduce the negative impact of disability on them, which is important to enhance their life satisfaction and prevent depressive symptoms.

## 1. Introduction

Depression is a common clinical mental disorder and is characterized by a persistent depressive mood [1,2], and it has become one of the most common medical illnesses [3,4]. It not only places a heavy burden on society because of long-term medicines and health services but also severely affects the health and quality of life of individuals [5,6]. A study reported that direct and indirect spending on treating major depression has been steadily increasing each year in the United States [7]. The prevalence of depressive symptoms was also quite common in China [8]. There were 2.2% of males and 3.3% of females in China suffering from major depressive disorders [9]. Wen et al. found that the incidence of depressive symptoms was as high as 22.3% through a 4-year follow-up among Chinese adults [10]. As such, it is essential to identify the factors related to depressive symptoms and probe into the mechanism among these factors.

As part of the aging process, increasing age is often accompanied by a decline in cognitive function [11], which is characterized by decreased memory, attention, and reasoning ability [12]. The link between cognitive function and depression has attracted a lot of attention, and there are many studies that have proven the relationship between them. The relationship between cognitive function and depression is bidirectional. That is, depression affects cognitive function, and, conversely, cognitive decline can also lead to depression. For example, depression can accelerate brain aging and increase the risk of cognitive impairment [13] through peripheral and cerebral microvascular dysfunction [14]. At the same time, studies have demonstrated that cognitive decline reduces people’s learning and thinking ability and then affects all aspects of life, work, and social interaction, which could increase their psychological stress and even lead to depression or other mental illnesses [15]. Tatiana et al. found that cognitive decline might predict depressive symptoms among older Hispanic adults living in the community [16]. Archana et al. used dynamic change models and potential difference scores to find that memory performance related to cognitive function predicted the changes in depression two years later [17]. By establishing the relationship between cognitive impairment and mood, Jennifer et al. found that participants with mild cognitive impairment had increased odds of depressive symptoms, but participants without cognitive impairment had no change in the rates of depressive symptoms [18]. In China, Yang et al. found that people with cognitive decline have a higher incidence of depression [19]. A cohort study has shown that participants with cognitive impairment had poorer mental status and an increased risk of depression one year later [20]. Clinical studies involving younger and elderly individuals have also established the inverse relationship between cognitive function and depression [21,22,23]. Moreover, in terms of gender differences, females in their mid-to-late 40s will go through menopause, which is the time of life when women in their mid-to-late 40s experience 12 consecutive months of amenorrhea because of a loss of follicular activity [24]. Due to the relative deficiency of androgens, estrogen, and progestin, postmenopausal women may experience depression and cognitive decline, which severely impairs postmenopausal females’ quality of life [25,26]. In consequence, it is also worth considering that the effect of cognitive function on depressive symptoms seems to differ by gender.

Although previous studies have explored the relationship between cognitive decline and depression, the impact of individual physical and psychological changes following cognitive decline on depression is also worthy of attention. As one of the adverse physical consequences of cognitive decline [27,28], disability can be considered as a series of physical limitations that influences individuals’ daily social, recreational, and work activities, which is generally measured by the activities of daily living (ADL) scale or the instrumental activities of daily living (IADL) scale. A cross-sectional study of elderly people in China indicated that nearly one in five individuals had difficulties with ADL disability but two in five had difficulties with IADL disability. Most elderly people need help with IADL, such as bathing and shopping [29]. IADL generally involve the more complex and varied activities of daily living compared with ADL, which require multiple cognitive domains and cognitive flexibility to complete together [30]. A study suggests that the association between cognitive function and ADL depends substantially on IADL [31]. Moreover, hippocampal and cortical gray matter volumes are correlated with IADL [32], suggesting that cognitive decline contributes to the incidence of IADL disability. According to a study involving 10,898 Chinese people, one of the most common risk factors for males regarding IADL disability was cognitive impairment [33]. Therefore, we chose IADL disability, which was more closely associated with cognitive function, as one of the indicators in this study. Regarding whether disability affects depressive symptoms in adults, prior studies have shown that compared with individuals without disabilities, individuals with disabilities were at increased risk of onset depression [34,35]. By constructing a Back Propagation neural network model, Chinese scholars found that disability ranked fourth among the risk factors of depression among Chinese individuals aged 45 or older [36]. These findings strongly suggest that disability is not just a consequence of cognitive decline but is also a key predictive factor for depression. In terms of IADL disability, previous research has confirmed that people with worse IADL performance were more likely to develop depressive symptoms over time [37]. A nationally representative study has shown that depressive symptoms were associated with an increase in IADL disability among Latinos [38]. In China, Li et al. found that IADL disability was significantly associated with an increased incidence of depression among older adults in both males and females [39]. Decreased ability of IADL may be a precursor of depression [40]. Therefore, it is of interest to explore the effect of IADL disability on the relationship between cognitive function and depressive symptoms.

Life satisfaction is a subjective judgment process, which is often considered a fundamental dimension for measuring individuals’ quality of life [41]. Among the studies on the relationship between cognitive function and life satisfaction, previous research has shown that elderly people with cognitive decline had lower life satisfaction [42]. A national study on 10,081 elderly South Koreans showed that cognitive function was an important factor in life satisfaction [43]. In a longitudinal study, life dissatisfaction was found to be related to the development of mild cognitive impairment among older adults [44]. However, there are few reports about the association between cognitive function and life satisfaction among Chinese people, which is worth exploring. In terms of the relationship between disability and life satisfaction, research has demonstrated that people with ADL and IADL disabilities were negatively associated with life satisfaction. The loss of independence for daily living abilities, especially for IADL ability, would trigger a significant decline in perceptions of quality of life and a lower level of life satisfaction [45]. In addition, life satisfaction has been proven to be linked to mental disorders, such as depression [42,46]. Zhang et al. studied nationally representative data in China and found that compared with those who were satisfied with their lives, the elderly with lower life satisfaction were more than twice as likely to be depressed [47]. Scholars have also found that cognitive decline was related to disability incidence, which was more common among elderly people who were dissatisfied with their lives [48]. It is concluded that cognitive function, IADL disability, and life satisfaction are related to each other. Given the relationship between cognitive function, life satisfaction, and depressive symptoms, life satisfaction may mediate the relationship between cognitive function and depressive symptoms.

Exploring the effect of IADL disability and life satisfaction on the relationship between cognitive function and depressive symptoms is conducive to a better understanding of the relationship between cognitive function and depressive symptoms and its internal mechanism, which also provides a reference for prevention and intervention for depression after cognitive decline. This study aimed to assess the relationships between cognitive function, IADL disability, life satisfaction, and depressive symptoms five years later among Chinese adults aged 40 years and above. We proposed three hypotheses for this study: H1, Cognitive function can have an impact on depressive symptoms five years later; H2, IADL disability and life satisfaction may have an independent mediating effect on the association between cognitive function and depressive symptoms five years later; and H3, IADL disability and life satisfaction would have a serial mediation effect between cognitive function and depressive symptoms five years later. We used data from three waves of the China Health and Retirement Longitudinal Study (CHARLS) that was conducted in 2013, 2015, and 2018, respectively, to empirically test the serial multiple mediating effects of IADL disability and life satisfaction between cognitive function and depressive symptoms five years later. At the same time, the influence of gender differences on this study was also considered.

## 2. Materials and Methods

### 2.1. Data and Study Design

The data were freely obtained from three waves of the China Health and Longitudinal Retirement Survey (CHARLS) conducted in 2013, 2015, and 2018. The CHARLS is a national longitudinal survey implemented by the National School for Development (China Center for Economic Research), which was first performed in 2011, and the participants have been followed up every two years [49]. The survey covers 28 provinces, 150 county-level units, and 450 communities in China, including information about Chinese adults, such as demographic background, family structure, socioeconomic status, and health behaviors [50].

We ascertained each participant’s cognitive function at baseline in 2013, his/her condition of IADL disability and life satisfaction in 2015, and his/her depressive symptoms in 2018. For the time frame, we excluded the participants who had already developed IADL disability, dissatisfaction with life, and depressive symptoms at baseline, as well as the participants with memory-related disorders, such as Alzheimer’s disease, brain atrophy, and Parkinson’s disease. Using data from each time frame, we evaluated the associations among cognitive function, IADL disability, life satisfaction, and depressive symptoms.

**Figure 1:**
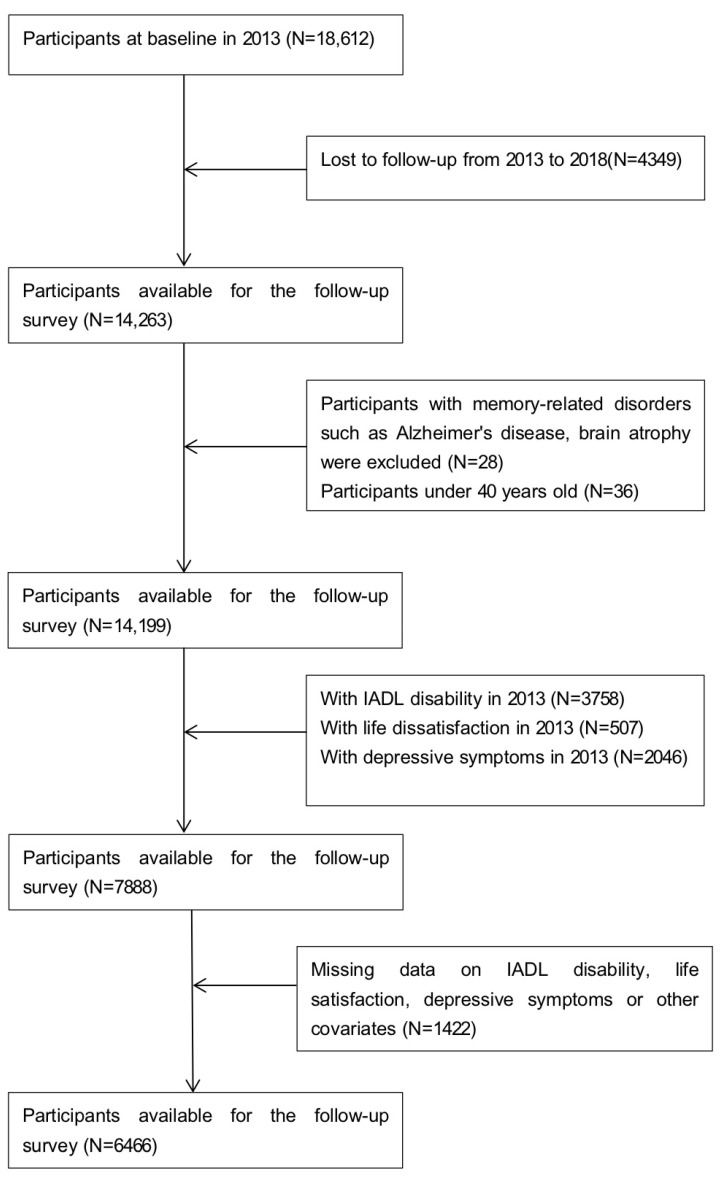
Flowchart of the inclusion of participants.

At baseline in 2013, the total sample consisted of 18,612 participants. We excluded 4349 individuals who were lost to follow-up from 2013 to 2018. Meanwhile, 28 participants were excluded due to memory-related disorders, such as Alzheimer’s disease, brain atrophy, and Parkinson’s disease, while 36 participants under 40 years old were also excluded. We further excluded those who had already developed IADL disability (n = 3758), life dissatisfaction (n = 507), or depressive symptoms (n = 2046) at baseline in 2013. Then, 1422 participants without complete information on the core variables, such as IADL disability and life satisfaction or other covariates, were also excluded. The final number of participants aged 40 years and above who were available for the follow-up survey was 6466. The details are shown in Figure 1.

### 2.2. Measurements

#### 2.2.1. Cognitive Function

The cognitive function in the CHARLS (2013) was assessed by the TICS-10 (orientation and attention), word recall (episodic memory), and figure drawing (visual-spatial abilities) [51]. The TICS (Telephone Interview of Cognitive Status) included the serial subtraction of 7 from 100 (up to five times), date (day, month, and year), day of the week, and season of the year. The scores of the TICS-10 ranged from 0 to 10. Word recall was used to assess episodic memory. After being shown 10 Chinese nouns, the participants were asked to recall as many words as they could immediately (immediate memory), in any order, and to recall them again four to ten minutes later (delayed recall). The episodic memory score includes the average number of immediate and delayed word recalls and ranged from 0 to 10. In terms of visuospatial ability, the respondents were shown a picture of two overlapped pentagons and asked to draw a similar figure. The participants received a score of 1 if they drew it correctly and no score otherwise [52,53]. The overall score ranged from 0 to 21, with higher scores indicating better cognitive function.

#### 2.2.2. IADL Disability

Disability in the instrumental activities of daily living (IADL) was described as dependence on at least one IADL task: doing housework, preparing meals, shopping, taking medication, managing money, and making a phone call [54]. The answers included 0 (no, I do not have any difficulty), 1 (I have difficulty but still can do it), 2 (yes, I have difficulty and need help), or 3 (I cannot do it). Those respondents were seen as dependent when they could not carry out the IADL scale activities independently (last three options) [55]. The total score ranged from 0 to 18, with higher scores indicating the more severe the dependence on the IADL item.

#### 2.2.3. Life Satisfaction

Life satisfaction was assessed by one broad question: “How satisfied were you about your life?” The respondents rated based on a 5-point Likert scale in which the higher scores indicated lower levels of life satisfaction. Assessing life satisfaction with an intuitive single question is easier to understand and accept, especially for older adults, which has been used in previous research [56,57].

#### 2.2.4. Depressive Symptoms

Depressive symptoms in the CHARLS were assessed by the 10-item short form of the Center for Epidemiologic Studies Depression Scale (CESD-10) [58]. Compared with the original CESD, the Chinese version of the CESD-10 also showed considerable accuracy in classifying the participants’ depressive symptoms (kappa = 0.84, *p* < 0.01) [58]. The CESD-10 comprised 10 questions about depression, and the answers included four options: 0 (rarely), 1 (some days; 1–2 days per week), 2 (occasionally; 3–4 days per week) and 3 (most of the time; 5–7 days per week) [59]. The total score ranged from 0 to 30, with a higher value indicating more depressive symptoms [60]. Individuals who scored more than 10 were identified as having depressive symptoms [61].

#### 2.2.5. Demographic Characteristics

We also considered the demographic characteristics of the individuals from the baseline in 2013, including age (years), gender (male, female), marital status (not married, married), smoking (yes, no), drinking (yes, no), social activities (yes, no), physical activities (yes, no), chronic disease (inapplicable, no, one, two and above), and self-rated health (very healthy, healthy, general, unhealthy, very unhealthy).

### 2.3. Data Analysis

In this study, IBM SPSS Statistics version 24 was employed for analysis and processing. We used descriptive analysis to describe the general characteristics of the study population. *t*-tests or chi-squared tests were applied to compare the group differences in gender. The PROCESS macro (Model 6) designed by Hayes [62] was used to examine whether IADL disability and life satisfaction mediated the association between cognitive function and depressive symptoms five years later. We also stratified the entire sample by sex to explore whether this relationship still existed. Based on bias-corrected bootstrapping with 5000 samples, we set the bootstrap confidence interval (CI) at 95%. Bootstrap intervals are considered to be significant when the 95%CI does not contain zero [63].

## 3. Results

### 3.1. Characteristics of Participants

As shown in Table 1, a total of 6466 participants aged 40 years or above were included in our study, and their mean age was 57.7 ± 8.5. The majority of the participants were married (92.8%), didn’t smoke (84.0%), and performed some physical activities (89.4%) or social activities (64.5%). A total of 7.6% of the participants clearly knew they had more than one chronic disease, and 40.4% of the participants drank. Among all the participants, only 6.9% and 15.9% had rated themselves as “very healthy” or “healthy”, respectively. In terms of gender, there were 3506 males and 2960 females, accounting for 54.2% and 45.8%, respectively. The mean age for the males was 58.8 ± 8.4 and for the females was 56.4 ± 8.3. The results of the *t*-tests or chi-squared tests showed that compared with the males, the females were more likely to be married, smoke, have lower than moderate self-rated health status, and were less likely to drink. More detailed demographic characteristics are shown in Table 1.

### 3.2. Correlation between the Core Variables

Correlation analysis revealed that cognitive function was negatively correlated with IADL disability (r = −0.214, *p* < 0.01) and depressive symptoms five years later (r = −0.152, *p* < 0.01). Cognitive function was positively correlated with life satisfaction (r = 0.026, *p* < 0.05). IADL disability (r = 0.152, *p* < 0.01) and life satisfaction (r = 0.147, *p* < 0.01) were positively correlated with depressive symptoms five years later. IADL disability (r = 0.039, *p* < 0.01) was positively correlated with life satisfaction (Table 2).

### 3.3. Mediating Effect Analyses

**Figure 2:**
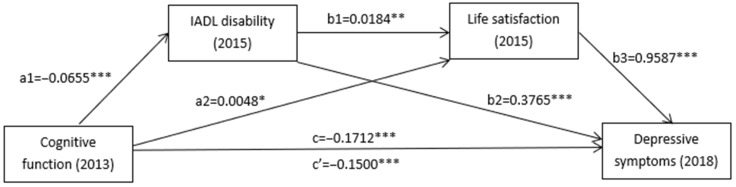
Serial mediation models for cognitive function, IADL disability, life satisfaction, and depressive symptoms. Note: Path coefficients were shown in the standardized regression coefficient. * *p* < 0.05, ** *p* < 0.01, *** *p* < 0.001.

To further elucidate the underlying mechanisms by which cognitive function is associated with depressive symptoms five years later, we explored the mediating roles of IADL disability and life satisfaction in this relationship. All the analyses in this study were conducted on the basis of adjusting for all the demographic characteristics. The analysis results are shown in Table 3 and Figure 2. Cognitive function had a significant and negative association with depressive symptoms five years later (*B* = −0.1712, 95%CI: −0.2050, −0.1374). Cognitive function had a significant and negative association with IADL disability (*B* = −0.0655, 95%CI: −0.0747, −0.0564). IADL disability had a significant and positive association with depressive symptoms five years later (*B* = 0.3765, 95%CI: 0.2874, 0.4655). Cognitive function had a significant and positive association with life satisfaction (*B* = 0.0048, 95%CI: 0.0003, 0.0094). Life satisfaction had a significant and positive association with depressive symptoms five years later (*B* = 0.9587, 95%CI: 0.7771, 1.1402). When controlling for IADL disability and life satisfaction, cognitive function was still negatively correlated with depressive symptoms five years later, although the coefficient decreased (*B* = −0.1500, 95%CI: −0.1839, −0.1161).

In addition, Table 3 presents the total and direct effects of cognitive function on depressive symptoms five years later and the mediating effect of IADL disability and life satisfaction. The results demonstrated that the total and direct effects of cognitive function on depressive symptoms five years later were −0.1712 and −0.1500, respectively. When IADL disability and life satisfaction were modelled as mediators, respectively, the path coefficients of cognitive function on depressive symptoms five years later indicated that IADL disability and life satisfaction had a significant mediating effect (Indirect effect1 = −0.0247, 95%CI: −0.0332, −0.0171; Indirect effect2 = 0.0046, 95%CI: 0.0000, 0.0094). In addition, IADL disability and life satisfaction played a serial mediating role in the association between cognitive function and depressive symptoms five years later (Indirect effect 3 = −0.0012, 95%CI: −0.0020, −0.0003). Therefore, three types of mediating effects were found in the relationship between cognitive function and depressive symptoms five years later: first, the mediating effect of IADL disability (effect = −0.0247); second, the mediating effect of life satisfaction (effect = 0.0046); and third, the serial mediating effect of IADL disability and life satisfaction (effect = −0.0012). All the results confirmed the hypothesis we made at the beginning of the study.

### 3.4. Gender Differences

With respect to gender differences, the full sample was divided into male (n = 3506) and female (n = 2960) groups for the mediating effect analyses. As shown in Table 3, IADL disability and life satisfaction partially mediated the relationship between cognitive function and depressive symptoms five years later for females. Additionally, the indirect roles of IADL disability and life satisfaction were also significant, respectively. However, for males, there is only one significant mediation path: cognitive function→ IADL disability→ depressive symptoms, which means that IADL disability was a mediator in the relationship between cognitive function and depressive symptoms five years later.

## 4. Discussion

Based on the national longitudinal dataset from CHARLS (2013, 2015, and 2018), we explored the relationship between cognitive function and depressive symptoms five years later among Chinese individuals aged 40 years and older and formulated a mediation model to examine the underlying mechanisms behind this specific association. The results showed that cognitive function is significantly associated with depressive symptoms five years later. In other words, cognitive decline is a risk factor for future depressive symptoms. Disability and life satisfaction play partial mediating roles and a serial mediation role in the relationship between cognitive function and depressive symptoms five years later.

The results suggested that cognitive function is significantly associated with subsequent depressive symptoms five years later, which is in accordance with previous studies [64,65]. This means that the worse the cognitive function, the higher the risk of depressive symptoms in the future. Several longitudinal studies have provided evidence that cognitive decline precedes the onset of depressive symptoms [66]. Clinically speaking, cognitive impairment has several pathophysiological mechanisms, such as disturbances in the hypothalamic–pituitary–adrenal axis and abnormalities in brain-derived neurotrophic signaling [67], which as risk factors might lead to increased chances of future depressive symptoms. In general, the risk of depression is most commonly diagnosed in relation to cognitive decline, such as memory lapses, slower thoughts, and confusion [68]. At the same time, people with cognitive decline experience depressive symptoms, which can be interpreted as a psychological response. In other words, depression can be conceptualized as a kind of psychological reaction to the perception of cognitive decline [69]. In addition, cognitive impairment may also make individuals more susceptible to cognitive distortions (e.g., unrealistic expectations, hyper-responsive to external stimulation), which can impair peoples’ regulatory emotions and further lead to depression [70,71].

After exploring the internal mechanism of the relationship between cognitive function and depressive symptoms five years later, we demonstrated that the indirect effect of this association can be mediated by IADL disability and life satisfaction, respectively. On the one hand, the results revealed that better baseline cognitive performance reduced the incidence of future IADL disability, which is consistent with previous findings that participants with impaired cognition were less likely to be independent [72,73]. A systematic review and meta-analysis established that IADL disability existed over a continuous course of cognitive decline [74]. Cognitive decline can affect people’s operational skills and fine control ability through neuropathological damage, resulting in IADL disability [75] and leading to losses of independence and productivity. When people become aware of the various adverse effects of cognitive decline on their daily life, such as the inconvenience of life and behaviors, it will break the psychological balance to cause many individuals obvious psychological burdens and will bring a series of depressive symptoms in the future [76]. On the other hand, life satisfaction played a mediating role between cognitive function and depressive symptoms. Interestingly, contrary to previous studies [77,78], we found that cognitive decline actually increased people’s life satisfaction, which in turn reduced the risk of developing depressive symptoms. With aging, there is a gradual decline in cognitive function among some people. Correspondingly, they may receive more material and emotional help from friends and relatives, which may prevent them from experiencing more negative emotions, improve their life satisfaction, and, thus, reduce the development of depressive symptoms [79]. Furthermore, the relevant policy guarantees and medical services for people with cognitive disorders provided by the government and departments also make them feel the care and support from society to a large extent [80,81], which will also improve their quality of life and life satisfaction to effectively prevent the occurrence of depression.

We also found that IADL disability and life satisfaction played partial mediating roles in the relationship between cognitive function and depressive symptoms five years later. In detail, baseline cognitive decline was significantly associated with future IADL disability and then reduced life satisfaction, which was in turn related to depressive symptoms in the future. Poor baseline cognitive ability increases the incidence of future IADL disability [27,82]. Adverse outcomes of IADL disability, such as social withdrawal, lack of energy/interest, and decreased self-efficacy, have been identified as strong predictors of reduced life satisfaction. Meanwhile, a large number of studies have shown that lower life satisfaction is an effective indicator of an individual’s exposure to significant depressive symptoms [83]. Compared with the general population, individuals with life dissatisfaction are more likely to have depressive symptoms and other mental health problems [84]. Therefore, IADL impairment caused by cognitive decline renders most adults unable to perform their social roles and daily life normally, thus affecting their life satisfaction [85]. To a certain extent, this will cause personal psychological distress that is difficult to adjust to and may even develop into depression in severe cases [86].

From the perspective of gender difference, our findings showed that IADL disability and life satisfaction played a chain mediating role between cognitive function and depressive symptoms five years later in females, while for males, only IADL disability had a significant mediating effect, which may be due to personality and biological differences between males and females. Moreover, menopause seems to expose women to the odds of cognitive impairment due to changes in sex hormone levels. The decline in cognitive function may interfere with an individual’s activities of daily living [87]. Increased sensitivity to hormonal changes in some menopausal women makes them more susceptible to the negative emotions associated with cognitive decline and IADL disability, leading to lower life satisfaction and an increased risk of depressive symptoms.

Finally, there are some limitations in this study. Firstly, the assessments of variables through self-report questions or a single item may have led to results bias and a lack of sensitivity [88], which made it difficult to detect subtle changes between the samples and also resulted in significant but very small correlations between some variables. In order to make the research results more convincing, future studies should try to introduce more objective and rich measurement methods to provide multidimensional information about the participants’ related indicators. Secondly, the dependent variable of depressive symptoms in this study was continuous. In order to explore the association between cognitive function and future depressive symptoms and its internal mechanism more clearly, clinical diagnosis results and more complex psychological tests should be combined, with the incidence of major depression as the endpoint for in-depth analysis. Thirdly, all the variables were collected through three waves of data from different years. This was neither a cross-sectional nor longitudinal study in nature, so in order to make the findings more convincing, longitudinal research should be conducted to analyze the changes regarding the relationship between cognitive function and future depressive symptoms over time and their causality.

## 5. Conclusions

This study provides evidence of the association between cognitive function and depressive symptoms five years later among Chinese individuals aged 40 years and older, and the support for the sequential mediating effects of IADL disability and life satisfaction between this relationship was confirmed. Future studies on this topic should reconsider and scrutinize in more depth the relationship between cognitive function and depressive symptoms while considering the differences in other factors. It is necessary to improve cognitive function and reduce the negative impact of disability on individuals, especially females, which is very important to enhance their life satisfaction and prevent depressive symptoms.

## Figures and Tables

**Table 1:** Demographic characteristics of the participants (n = 6466).

Variables	Category	Total (n = 6466)	Males (n = 3506)	Females (n = 2960)	χ^2^ or t Statistics	*p* Value
Age (M ± SD)		57.7 ± 8.5	58.8 ± 8.4	56.4 ± 8.3	11.433	0.834
Marital status	Not married	466 (7.2)	198 (5.6)	268 (9.1)	27.851	<0.001
	Married	6000 (92.8)	3308 (94.4)	2692 (90.9)		
Chronic disease	Inapplicable	5595 (86.5)	3051 (87.0)	2544 (85.9)	4.195	0.241
	No	380 (5.9)	187 (5.3)	193 (6.5)		
	One	258 (4.0)	139 (4.0)	119 (4.0)		
	Two and above	233 (3.6)	129 (3.7)	104 (3.5)		
Smoking	No	5429 (84.0)	2555 (72.9)	2874 (97.1)	699.152	<0.001
	Yes	1037 (16.0)	951 (27.1)	86 (2.9)		
Drinking	No	3852 (59.6)	1334 (38.0)	2518 (85.1)	1473.280	<0.001
	Yes	2614 (40.4)	2172 (62.0)	442 (14.9)		
Physical activities	No	5782 (89.4)	3150 (89.8)	2632 (88.9)	1.458	0.227
	Yes	684 (10.6)	356 (10.2)	328 (11.1)		
Social activities	No	2294 (35.5)	1233 (35.2)	1061 (35.8)	0.321	0.571
	Yes	4172 (64.5)	2273 (64.8)	1899 (64.2)		
Self-rated health status	Very healthy	447 (6.9)	257 (7.3)	190 (6.4)	17.072	0.002
	Healthy	1027 (15.9)	579 (16.5)	448 (15.1)		
	General	2404 (37.2)	1329 (37.9)	1075 (36.3)		
	Unhealthy	2153 (33.3)	1139 (32.5)	1014 (34.3)		
	Very unhealthy	435 (6.7)	202 (5.8)	233 (7.9)		

**Table 2:** Correlations among cognitive function, IADL disability, life satisfaction, and depressive symptoms.

Variables	Cognitive Function	IADL Disability	Life Satisfaction	Depressive Symptoms
Cognitive function	1			
IADL disability	−0.214 **	1		
Life satisfaction	0.026 *	0.039 **	1	
Depressive symptoms	−0.152 **	0.152 **	0.147 **	1

Note: ** *p* < 0.01 (two-tailed). * *p* < 0.05 (two-tailed).

**Table 3:** Hypothesized serial mediation model of cognitive function, IADL disability, life satisfaction, and depressive symptoms.

	Pathway	B	SE	BootLLCI	BootULCI
Total samples	Total effect (c)	−0.1712	0.0173	−0.2050	−0.1374
	Direct effect (c’)	−0.1500	0.0173	−0.1839	−0.1161
	a1	−0.0655	0.0047	−0.0747	−0.0564
	a2	0.0048	0.0023	0.0003	0.0094
	b1	0.0184	0.0061	0.0064	0.0303
	b2	0.3765	0.0454	0.2874	0.4655
	b3	0.9587	0.0926	0.7771	1.1402
	Indirect effects				
	Total indirect effects	−0.0212	0.0048	−0.0311	−0.0121
	Indirect 1	−0.0247	0.0041	−0.0332	−0.0171
	Indirect 2	0.0046	0.0024	0.0000	0.0094
	Indirect 3	−0.0012	0.0004	−0.0020	−0.0003
Males	Total effect (c)	−0.1561	0.0245	−0.2042	−0.1080
	Direct effect (c’)	−0.1307	0.0244	−0.1785	−0.0828
	a1	−0.0552	0.0062	−0.0673	−0.0431
	a2	0.0025	0.0034	−0.0042	0.0092
	b1	0.0085	0.0093	−0.0097	0.0267
	b2	0.4951	0.0662	0.3653	0.6250
	b3	0.9295	0.1205	0.6932	1.1658
	Indirect effects				
	Total indirect effects	−0.0254	0.0066	−0.0384	−0.0127
	Indirect 1	−0.0273	0.0056	−0.0388	−0.0168
	Indirect 2	0.0024	0.0033	−0.0042	0.0092
	Indirect 3	−0.0004	0.0005	−0.0015	0.0006
Females	Total effect (c)	−0.1853	0.0245	−0.2334	−0.1372
	Direct effect (c’)	−0.1688	0.0247	−0.2172	−0.1203
	a1	−0.0724	0.0071	−0.0862	−0.0585
	a2	0.0071	0.0032	0.0009	0.0134
	b1	0.0268	0.0081	0.0109	0.0427
	b2	0.2989	0.0634	0.1746	0.4233
	b3	0.9838	0.1435	0.7025	1.2652
	Indirect effects				
	Total indirect effects	−0.0165	0.0068	−0.0296	−0.0033
	Indirect 1	−0.0216	0.0057	−0.0331	−0.0111
	Indirect 2	0.0070	0.0035	0.0006	0.0143
	Indirect 3	−0.0019	0.0007	−0.0035	−0.0006

Abbreviation: Indirect 1, Cognitive function → IADL disability → Depressive symptoms; Indirect 2, Cognitive function → Life satisfaction → Depressive symptoms; Indirect 3, Cognitive function → IADL disability →Life satisfaction → Depressive symptoms.

## Data Availability

The raw data used in this study are freely available from the China Health and Retirement Longitudinal Study (CHARLS; http://charls.pku.edu.cn/, accessed on 3 May 2022). Researchers are required to apply for permission to use the data.
